# A 3D–Predicted Structure of the Amine Oxidase Domain of Lysyl Oxidase–Like 2

**DOI:** 10.3390/ijms232113385

**Published:** 2022-11-02

**Authors:** Alex A. Meier, Krzysztof Kuczera, Minae Mure

**Affiliations:** 1 Department of Chemistry, The University of Kansas, Lawrence, KS 66045, USA; 2 Department of Molecular Biosciences, University of Kansas, Lawrence, KS 66045, USA

**Keywords:** lysyl oxidase–like 2, lysine tyrosylquinone, molecular modeling, molecular dynamic simulation

## Abstract

Lysyl oxidase–like 2 (LOXL2) has been recognized as an attractive drug target for anti–fibrotic and anti–tumor therapies. However, the structure–based drug design of LOXL2 has been very challenging due to the lack of structural information of the catalytically–competent LOXL2. In this study; we generated a 3D–predicted structure of the C–terminal amine oxidase domain of LOXL2 containing the lysine tyrosylquinone (LTQ) cofactor from the 2.4Å crystal structure of the Zn^2+^–bound precursor (lacking LTQ; PDB:5ZE3); this was achieved by molecular modeling and molecular dynamics simulation based on our solution studies of a mature LOXL2 that is inhibited by 2–hydrazinopyridine. The overall structures of the 3D–modeled mature LOXL2 and the Zn^2+^–bound precursor are very similar (RMSD = 1.070Å), and disulfide bonds are conserved. The major difference of the mature and the precursor LOXL2 is the secondary structure of the pentapeptide (His652–Lys653–Ala654–Ser655–Phe656) containing Lys653 (the precursor residue of the LTQ cofactor). We anticipate that this peptide is flexible in solution to accommodate the conformation that enables the LTQ cofactor formation as opposed to the β–sheet observed in 5ZE3. We discuss the active site environment surrounding LTQ and Cu^2+^ of the 3D–predicted structure.

## 1. Introduction

The aberrant expression of lysyl oxidase–like 2 (LOXL2) is associated with fibrotic disorders and metastatic/invasive tumors, and LOXL2 has shown to promote activation of fibroblasts [1,2], proliferation and metastasis/invasion of tumor cells [3,4,5,6], and tumor angiogenesis [7,8]. LOXL2 is a Cu^2+^–dependent amine oxidase that catalyzes the oxidative deamination of the ϵ–amino group of lysines and hydroxylysines of ECM proteins such as collagen type IV and tropoelastin [9,10]. Recently, platelet growth factor receptor β (PDGFRβ) has been identified as a new class of LOXL2 substrate, where tumor cell–secreted LOXL2 together with platelet derived growth factor (PDGF)–AB have been shown to activate PDGFRβ in fibroblasts to promote cell proliferation via activation of the ERK signaling pathway [11]. In that study, an irreversible inhibitor for LOXL2, PXS–S1C (Pharmaxis Cooperation LLC, Sydney, NSW, Australia), was shown to attenuate fibroblasts proliferation, activation of PDGFRβ (phosphorylation), and ERK activation. In addition to PXS–S1C, several small molecule inhibitors were synthesized and shown both to inactivate LOXL2 (IC_50_ = 0.008–0.203 μM) and to repress the epithelial–to–mesenchymal transition (EMT), proliferation of tumor cells and progression of fibrosis [12,13,14,15,16,17].

**Figure 1:**
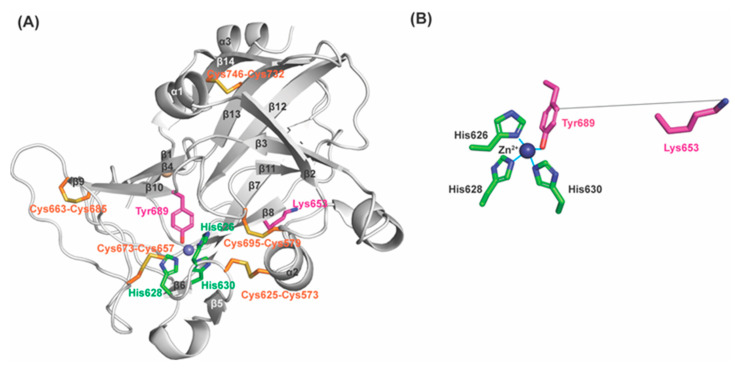
The 2.4Å structure of a Zn^2+^–bound precursor LOXL2 (PDB: 5ZE3). (**A**) The amine oxidase domain of LOXL2 and precursor residues (Lys653 and Tyr689 in magenta) of the LTQ cofactor and Zn^2+^ (navy sphere) occupies the predicted Cu^2+^–binding site, His626–X–His628–X–His630 motif (in green). (**B**) Lys653 is located 16.6Å distance from Tyr689 (in solid grey line). Zn^2+^ is in tetrahedral coordination geometry.

LOXL2 contains the lysine tyrosylquinone (LTQ) cofactor that is post–translationally derived from Lys653 and Tyr689 in the C–terminal catalytic domain [18,19]. Based on the mechanism of the topaquinone (TPQ) cofactor biogenesis in copper amine oxidases (CAOs), it has been proposed that a 1,4–addition (Michael addition) of the ϵ–amino group of Lys653 to dopaquinone (DPQ) intermediate derived from Tyr689, followed by O_2_–oxidation yields LTQ [20,21]. Development of LOXL2–targeted therapy has been very challenging due to the lack of recombinant LOXL2 (rLOXL2) suitable to conduct mechanistic and structure–function correlation studies. Currently, a 2.4Å structure of a precursor Δ1–2SRCR–LOXL2 (in which the first two of the four SRCR domains at the N–terminus of the full–length LOXL2, fl–LOXL2, are truncated, Figure 1A) without LTQ is available (PDB:5ZE3) [22]. In 5ZE3, Zn^2+^ occupies the proposed Cu^2+^–binding site (His626–X–His628–X–His630) and the precursor residues of LTQ (Lys653 and Tyr689) are seen 16.6Å apart (Figure 1B). Thus, a substantial conformational rearrangement (including the possibility of disulfide shuffling) has been proposed to take place during LTQ biogenesis [22,23].

**Figure 2:**
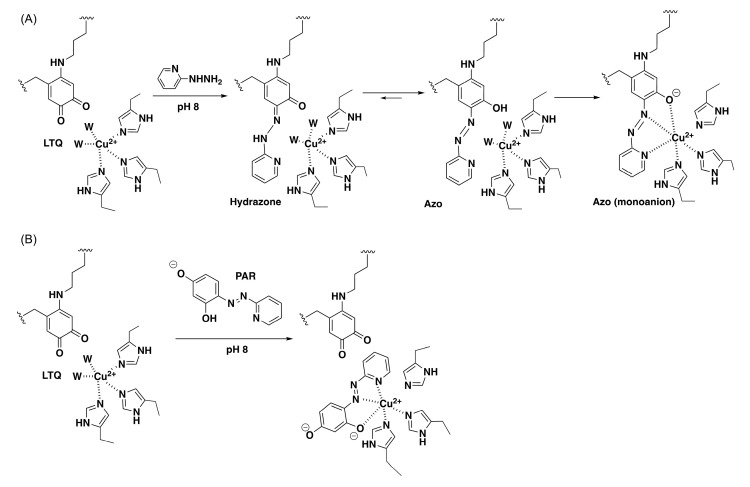
The LTQ cofactor resides within 2.9Å of the active site Cu^2+^ in the catalytically–competent LOXL2 [25]. (**A**) 2HP covalently modifies the C5 carbonyl group of LTQ to form LTQ–2HP adduct. Ligation of LTQ–2HP adduct to the active site Cu^2+^ through O4 results in the formation of the monoanion form of LTQ–2HP (λ_max_ = 531 nm) at pH 8.0. (**B**) The active site Cu^2+^ in the mature LOXL2 can be titrated by PAR. W: water ligand to Cu^2+^.

Previously, we have characterized biophysical and biochemical properties of recombinant fl–LOXL2 and Δ1–2SRCR–LOXL2 containing the nearly stoichiometric amount of LTQ (≤95%). Hydrodynamic radii and radii of gyration of the mature LOXL2 (containing LTQ) are very similar to the values that were calculated from the 3D–predicted structure of fl–LOXL2 by AlphaFold 2 (https://alphafold.ebi.ac.uk/entry/Q9Y4K0; accessed on 1 June 2022) and the Zn^2+^–bound precursor Δ1–2SRCR–LOXL2 [24]. These results suggest that the overall structures of the precursor and the mature LOXL2 are very similar. Subsequently, we conducted mass spectrometry–based disulfide mapping of the precursor LOXL2 and the mature LOXL2 that was inhibited by 2–hydrazinopyridine (2HP) and demonstrated that the cysteine–pairing pattern of disulfide bonds is unaltered before and after LTQ biogenesis [19]. These results support our hypothesis that a substantial conformational rearrangement is not required for LTQ biogenesis. Further, the UV–vis and resonance Raman spectroscopic features of 2HP–inhibited LOXL2 in comparison to the corresponding model compounds strongly suggested that 2HP covalently modifies LTQ (LTQ–2HP), and that LTQ–2HP is ligated to the active site Cu^2+^ (Figure 2A) [25], analogous to the 2HP–modified topaquinone (TPQ) cofactor (TPQ–2HP) detected in a crystal structure of Y369F–ECAO (a CAO from *Escherichia coli*, *E. coli*) (Figure S1A) [26]. Interestingly, LTQ–2HP contains the structural motif similar to 4–(2–pyridylazo)resorcinol (PAR), a known chelator. We found that the active site Cu^2+^ in the mature LOXL2 can be fully titrated by PAR (Figure 2B) [25]. Based on these results, we believe that the LTQ cofactor resides within 2.9Å from the active site Cu^2+^ in the mature LOXL2 and both LTQ and Cu^2+^ are solvent–exposed as in the case of the precursor Tyr689 and Zn^2+^ in the precursor. We looked into the Zn^2+^–bound precursor structure of Δ1–2SRCR–LOXL2 and modeled LTQ–2HP into the precursor structure via molecular modeling and molecular dynamics (MD) simulation. We then edited LTQ–2HP to LTQ to generate the resting form of the mature LOXL2. The resulting structure gives the first insight into the active site environment of the mature form of LOXL2.

## 2. Results

### 2.1. Factors That May Stabilize the Unproductive Conformation of Lys653 in the Precursor Structure

**Figure 3:**
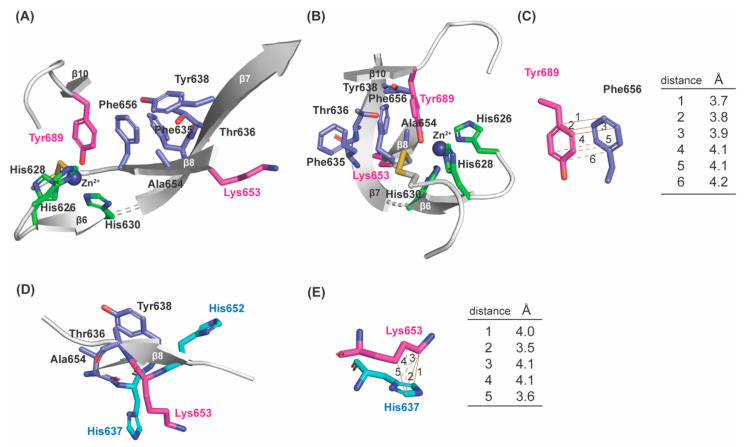
A hydrophobic patch is found in the active site of the precursor LOXL2 structure (PDB: 5ZE3). (**A**) Phe635, Thr636, Tyr638, Ala654, and Phe656 comprise a hydrophobic patch and that seems to stabilize the short β–sheet (β8) in an unproductive conformation. Consequently, Lys653 is in the orientation to face away from Tyr689 to prevent the LTQ formation. (**B**) The same structure as (**A**) from a different angle. (**C**) Tyr689 and Phe656 are in π–π stacking interaction and within Van der Waals radii. (**D**) and (**E**) In addition, Lys653 is in hydrophobic interaction with His637.

**Figure 4:**
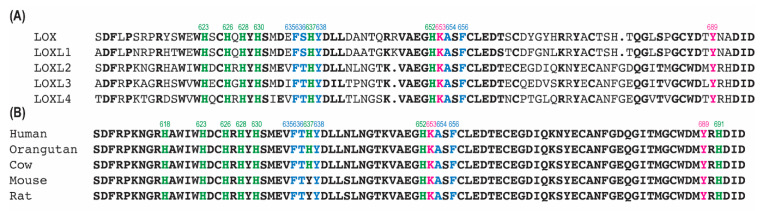
Amino acid sequence alignment of (**A**) human LOX–family of proteins and (**B**) mammalian LOXL2s. The precursor residues (Lys653 and Tyr689) of LTQ are in pink, His residues are in green and conserved His residues are in bold. Residues that comprise the hydrophobic patch (Figure 3) are in blue. Numbering of residues are that of human LOXL2. Multiple sequence alignment was conducted by COBALT (https://www.ncbi.nlm.nih.gov/tools/cobalt/re_cobalt.cgi).

In the Zn^2+^–bound precursor structure (Figure 1A), Tyr689 is at the C–term of β10 which is antiparallel to β4; Lys653 is on β8 (His653–Lys653–Ala654–Ser655–Phe656) which is antiparallel to β7 (Phe635–Thr636–Hys637–Tyr638–Asp639–Leu640); and three His residues of the Cu^2+^–binding motif (His626–X–His628–X–His630) are on a short loop and the short β6. Besides these secondary structures, the rest are all loops. To enable 1,4–Michael addition of the ϵ–amino group of Lys653 to C2 of DPQ689 to form LTQ, some structural (backbone) flexibility of Lys653 is anticipated. Upon a careful inspection of the precursor structure, we noticed a hydrophobic patch that is composed of five residues (Phe635, Thr636, Tyr638, Ala654 and Phe656) adjacent to Tyr689, and Phe656 is in π–π stacking interaction with Tyr689 (Figure 3A–C). Among the human LOX–family of proteins, all except Thr636 are completely conserved (Ser in LOX and LOXL1) (Figure 4A). Among the mammalian LOXL2 family, these five residues are completely conserved (Figure 4B). Lys653 faces away from Tyr689 (16.6Å apart) and Lys653 (Cϵ) is in Van der Waals interaction with His637 (Cδ2, Cϵ1, and Cγ) on β7 (Figure 3D,E). We believe that the π–π stacking of Tyr689 and Phe656 and the Van der Waals interaction of Lys653 and His637 are most likely consequences of Zn^2+^–binding to the Cu^2+^–binding site. Since Zn^2+^ cannot support the activation/oxidation of Tyr689, the unproductive conformation of Lys653 seems to be thermodynamically favored during protein crystallization. However, our solution study of the catalytically–competent LOXL2 strongly indicates that β8 and the Lys653 sidechain are not fixed in the unproductive conformation seen in the precursor structure: instead, they can accommodate the productive conformation of Lys653 to support the LTQ cofactor biogenesis in the presence of Cu^2+^ under aerobic conditions [19,24].

### 2.2. Prediction of the Active Site Structure of 2HP–Inhibited LOXL2 by Molecular Modeling and Energy Minimization

**Figure 5:**
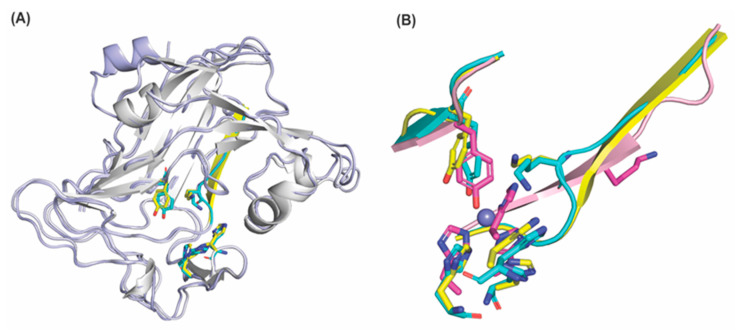
Overlay of the amine oxidase domains of LOX and LOXL2. (**A**) Overlay of AlphaFold 2–predicted human LOX (peptide backbone: in white; the LTQ precursors in yellow, His292-X-His294-X-His296: in yellow) and mouse LOXL2 (peptide backbone: in light blue; the LTQ precursors: in cyan, His628, His630, His632: in cyan). The major difference is the secondary structure of the heptapeptide (Val315–Ala321 in human LOX: β–sheet in yellow and Val650–Ala656 in mouse LOXL2: loop in cyan). In both structures, the precursor Lys residues (Lys320 in human LOX and Lys655 in mouse LOXL2) are within 4.0Å and 4.7Å from the C2 position of the precursor Tyr residues (Tyr355 in human LOX and Tyr691 in mouse LOXL2), respectively. (**B**) Overlay of the precursor Lys–containing heptapeptides, the precursor Tyr–containing peptides and His–X–His–X–His of the Zn^2+^–bound precursor LOXL2 (PDB: 5ze3, in light pink, residues are in magenta, Zn^2+^ is in slate), AlphaFold 2–prediced human LOX (in yellow) and AlphaFold2–predicted mouse LOXL2 (in cyan).

During the course of this study, 3D–predicted structures of the human LOX–family of proteins were generated by AlphaFold 2 [27,28]. Among them, LOX (https://alphafold.ebi.ac.uk/entry/A0A7P0SNB0: accessed on 1 June 2022) contains a longer β–sheet (Val315–Ala316–Glu317–Gly318–His319–Lys320–Ala321) corresponding to the loop (Val648–Ala649–Glu650–Gly651) and β8 in the precursor LOXL2, that is antiparallel to β7 (Phe301–Asp308). Interestingly, this longer β–sheet in LOX is in the productive conformation for the LTQ cofactor biogenesis, where the ϵ–side chain of Lys320 (Lys653 in LOXL2) is within 4Å of Tyr355 (Tyr689 in LOXL2) (Figure 5A, in yellow). Although this conformation was not seen for the AlphaFold 2–predicted structures of LOXL2 (https://alphafold.ebi.ac.uk/entry/W6I206: accessed on 1 June 2022) and other members of the human LOX family of proteins, the similar conformation is detected in AlphaFold 2–predicted mouse LOXL2 (https://alphafold.ebi.ac.uk/entry/Q5PR71: accessed on 1 June 2022) (Figure 5A, in cyan) [24]. We first manually applied the dihedral angles of the hexapeptide (His319–Lys320–Ala321–Ser322–Phe323–Cys324) of LOX (Figure 5A, in yellow) to the corresponding hexapeptide (His652–Lys653–Ala654–Ser655–Phe656–Cys657) in the LOXL2 precursor structure (Figure 1A and Figure 3A). As a result, the ϵ–amino group of Lys653 became closer (~6.5Å) to C2 of Tyr689.

**Figure 6:**
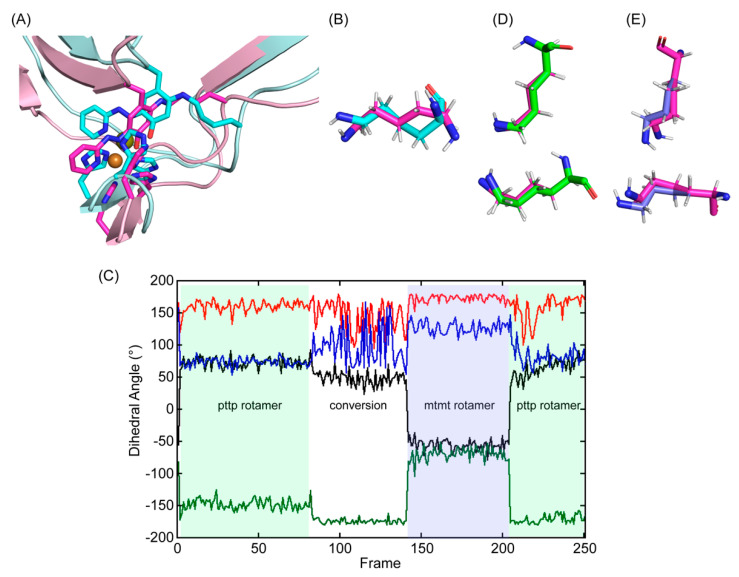
Molecular modeling and energy minimization of 2HP–inhibited Δ1–2SRCR–LOXL2 in comparison to the structure after MD–simulation. (**A**) The initial 3D–model (in cyan) was generated by replacing Tyr689 with DPQ–2HP and crosslinking C2 with the ϵ–amino side chain of Lys653. The single bond between ϵ–nitrogen and δ–carbon of Lys653 and the aromatic ring of Tyr698 (LTQ–2HP) was not on the same plane (dihedral angle = 15°) and the –(CH_2_)_4_–NH– side chain of Lys653 was in a higher energy conformation. After manual rearrangement of the hexapeptide (His652–Lys653–Ala654–Ser655–Phe656–Cys657) and subsequent MD–simulation, the –(CH_2_)_4_–NH– side chain of Lys653 was in a more relaxed conformation (in magenta and light pink). (**B**) An overlaid view of Lys653 in the initial 3D–model (in cyan) and in the final structure after MD–simulation (in magenta) (**C**) Dihedral angles of Lys653 during the 10 ns MD–simulation (40 ps/frame): Χ1 (dihedral angle between N–Cα–Cβ–Cγ) in black; X2 (dihedral angle between Cα–Cβ–Cγ–Cδ) in red; X3 (dihedral angle between Cβ–Cγ–Cδ–Cϵ) in green; X4 (dihedral angle between Cγ–Cδ–Cϵ–Nζ) in blue. We observed pttp and mtmt rotamers among the ideal rotamers reported for Lys653 in the penultimate rotamer library: t stands for the side chain dihedral angles of 180°, p stands for the side chain dihedral angles of 65°, and m stands for the side chain dihedral angles of – 65° [31]. (**D**) An overlaid view of Lys653 after MD–simulation (in magenta) with the pttp rotamer (in green) (**E**) An overlaid view of Lys653 after MD–simulation (in magenta) with the mtmt rotamer (in slate).

Tyr689 was then replaced with TPQ–2HP using the coordinates and dihedral angles of that detected in the X–ray crystal structure of Y369F–ECAO (Figure S1A) [26]. The C2 oxygen atom of TPQ–2HP (Tyr689) was removed to generate DPQ689 and was covalently modified with 2HP (DPQ–2HP) and then the ϵ–amino group of Lys653 was manually crosslinked to the C2 of DPQ–2HP to generate LTQ–2HP via a usage of the patch function of CHARMM [29]. The parameters for the patch residue were first predicted by CHARMM General Force Field (CGenFF), based on the X–ray crystal structure of the corresponding Cu^2+^–bound LTQ–2HP model compound (Figure S1B) [25,30]. While the structure of LTQ–2HP was successfully engineered in LOXL2, the –(CH_2_)_4_–NH– side chain of Lys653 was forced to adopt an unfavorable rotamer in higher energy (Figure 6A). Subsequently, the coordinates of the aforementioned hexapeptide in 3D–modeled LOX were manually adjusted to allow Lys653 to adopt a stable conformation, the pttp rotamer in the penultimate rotamer library (Figure 6B,D) [31].

### 2.3. Molecular Dynamics (MD)–Simulation of the Predicted Structure of 2HP–Inhibited LOXL2

After generating the 3D–predicted structure of 2HP–inhibited LOXL2, the predicted structure was submitted for 10 ns MD–simulation through GROMACS [32,33]. During the production phase of the simulation, the simulated structure was comparable to the initial structure (RMSD = 1.41Å) (Figure 6A). For the most of frames during MD–simulation, Lys653 remained in a slightly distorted pttp rotamer (frames 1–80 and 208–251) (Figure 6C, in green shade; Figure 6D, in green). There was a period halfway through the simulation where Lys653 was more mobile (frames 81–145), and this led to a conversion of the pttp rotamer to a slightly distorted mtmt rotamer (frames 146–207) (Figure 6C, in slate shade; Figure 6E in slate). Lys653 then quickly converted back to the pttp rotamer (frames 208–251) (Figure 6C). These results indicate that the active site has some space to accommodate Lys653 to adopt at least two different stable rotamers while being crosslinked to DPQ689.

**Figure 7:**
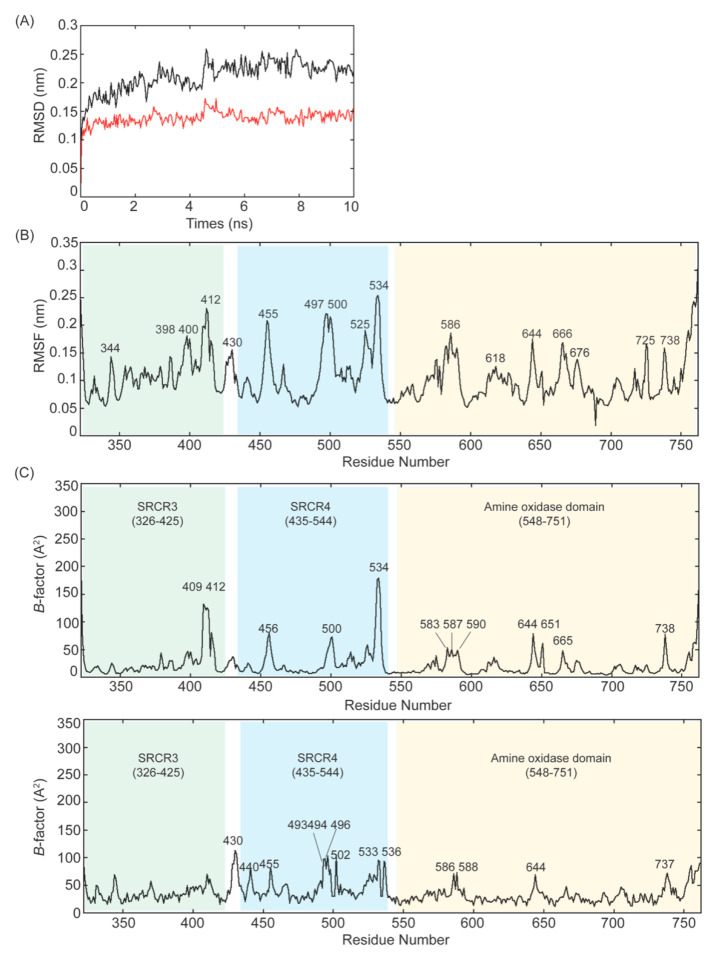
MD–simulation of the 2HP–inhibited Δ1–2SRCR–LOXL2. (**A**) Plot of RMSD values of the Cα atoms of Δ1–2SRCR–LOXL2 backbone (in black) and the amine oxidase domain of LOXL2 (in red) during 10 ns of simulation. The MD–simulation for the former shows a gradual increase in the RMSD value with fluctuations, stabilizing at an average of 0.23 nm, while the latter quickly (≤1 ns) reaches a plateau of RMSD, stabilizing at an average of 0.14 nm. (**B**) Plot of RMSF values of the Cα atoms of Δ1–2SRCR–LOXL2 for 10 ns of simulation where fluctuations are ≤0.253 nm. (**C**) Top panel: Cα *B*–factor/residue of the MD–simulated Δ1–2SRCR–LOXL2 plotted against amino acid residue numbers. All peaks except the last peak at 755 (α3 helix) are loops. Bottom panel: Cα *B*–factor/residues of precursor LOXL2 plotted against amino acid residue numbers.

The frequency of the pttp rotamer in frames suggests that it is the most stable rotamer, therefore we chose the pttp rotamer for visualization of the MD–simulated final structure. Root–mean–square–deviation (RMSD) is a standard measure of the average distance between backbones from the initial conformation to the final conformation. In our MD–simulation, both RMSD values for Δ1–2SRCR–LOXL2 (in black) and the amine oxidase domain (in red) are relatively consistent over 10 ns, especially between 6–10 ns (Figure 7A). The former has a slightly higher RMSD value (0.229 ± 0.012 nm) than the latter (0.141 ± 0.008 nm), suggesting that there is some degree of deviation from the original state. However, a configuration with an RMSD value ≤0.25 nm is considered very close to the original state [34], suggesting that neither form of LOXL2 undergoes significant conformational change. The structural fluctuations in the MD trajectory can be measured by root–mean–square–fluctuation (RMSF) of Cα carbons. RMSF quantifies the difference in structures over time and reveals which parts in the protein structure are the most mobile. We obtained the maximum, minimum and average RMSF values (0.3131, 0.0186, 0.105 ± 0.042 nm) (Figure 7B) and the low average RMSF indicates each Cα atom exhibiting stability during MD–simulation. Almost all peaks in Figure 7B correspond to loop regions except the peak at the residue 755 which corresponds to α3. The *B*–factor (Debye–Waller factor) or atomic displacement parameter, proportional to atomic mean–square fluctuations, is used to describe attenuation of X–ray scattering caused by thermal motion. Figure 7C shows plots for Cα *B*–factor/residue against residue numbers (top panel: MD–simulated Δ1–2SRCR–LOXL2, bottom panel: precursor LOXL2). Peaks in the *B*–factor plot are considered as flexible regions and the height of the respective peak is the relative degree of flexibility.

### 2.4. Principle Component Analysis (PCA) and Addison Parameter (τ) Calculation of 2HP–Inhibited Δ1–2SRCR–LOXL2

**Figure 8:**
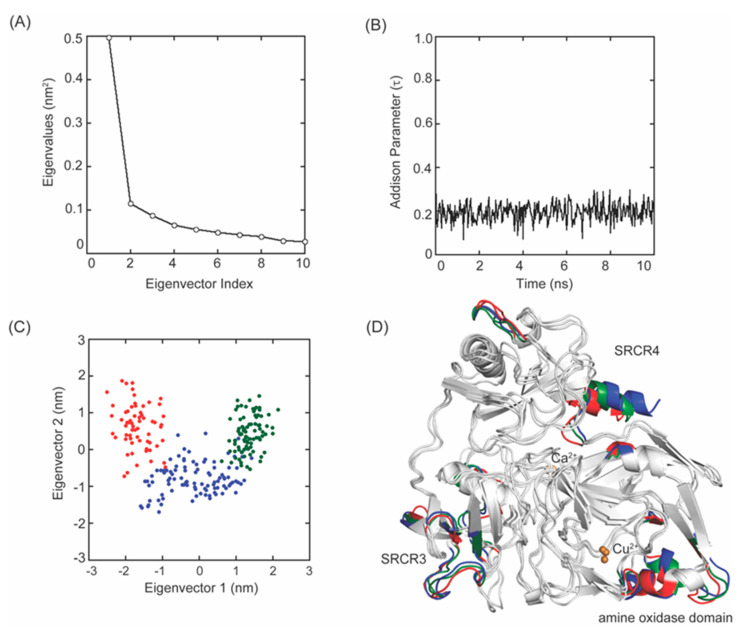
MD–simulation of the 2HP–inhibited Δ1–2SRCR–LOXL2. (**A**) A plot of eigenvalues versus the first 10 eigenvector indices obtained from the covariance matrix of Cα atoms over a stable trajectory of 10 ns of MD–simulation. (**B**) A plot of the Addison parameter (τ) values of the active site Cu^2+^ during 10 s MD–simulation indicates a square pyramidal coordination geometry. (**C**) Projection of the motion of Cα atoms of Δ1–2SRCR–LOXL2 along the first two principal eigenvectors during 0–1.08 ns (in red), 1.12–4.96 ns (in blue), and 5–10 ns (in green). (**D**) The Cα atoms corresponding to those in (**C**) are highlighted in the same colors.

**Figure 9:**
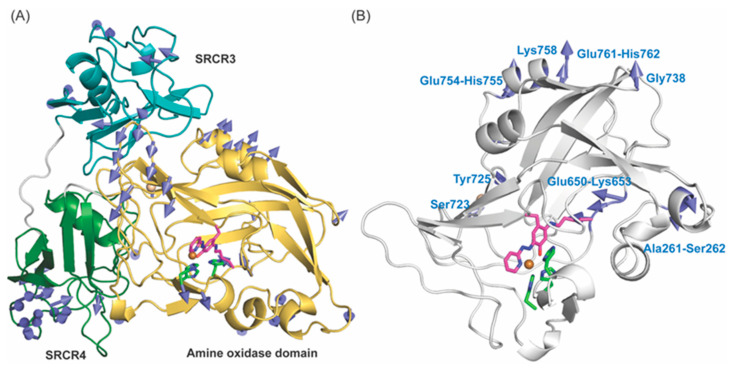
Displacement of the Cα atoms in 2HP–inhibited Δ1–2SRCR–LOXL2 (**A**) and the amine oxidase domain of 2HP–inhibited LOXL2 (**B**) where regions of peptides/amino acids exceeding 3Å (**A**) or 2.5Å (**B**) are visualized by vectors (in teal). In (**A**), SRCR3 and SRCR4 domains are in cyan and in green, respectively, and the amine oxidase domain is in yellow. The structures shown are in the very first phase and vectors indicating the difference between the first and the last phase of MD–simulation.

PCA is a statistical technique that reduces the number of dimensions to extract essential elements in data using a covariance matrix [35]. PCA projects the trajectory of a MD–simulation and isolates the dominant modes in protein motion. Eigenvectors and eigenvalues calculated from the covariance matrix represent directions and magnitudes of fluctuations in the Cα atomic coordinates which enables us to estimate degrees of flexibility of peptide backbones [36]. For 2HP–inhibited LOXL2, the first three eigenvalues account for 32.2%, 7.5% and 5.6% of the total atomic fluctuations in the 10 ns MD–simulation (Figure 8A). Since the first few eigenvectors play an essential role in overall motions, we considered the first two eigenvectors (eigenvectors 1 and 2) to represent our PCA analysis. The MD–simulation produced trajectories of Cα atomic positions as a function of time and provided three representative stable states (Figure 8C,D). The analysis of *B*–factors indicates that some fluctuations observed during our MD–simulation of Δ1–2SRCR–LOXL2 stem from the movement of surface exposed loops (Figure 8D and Figure 9A,B). Three His residues comprising the Cu^2+^–binding site (in green) as well as LTQ–2HP (in magenta) remained at constant distances to Cu^2+^ throughout the simulation (Figure 9A,B). These results indicate that the active site structure is relatively stable.

**Figure 10:**
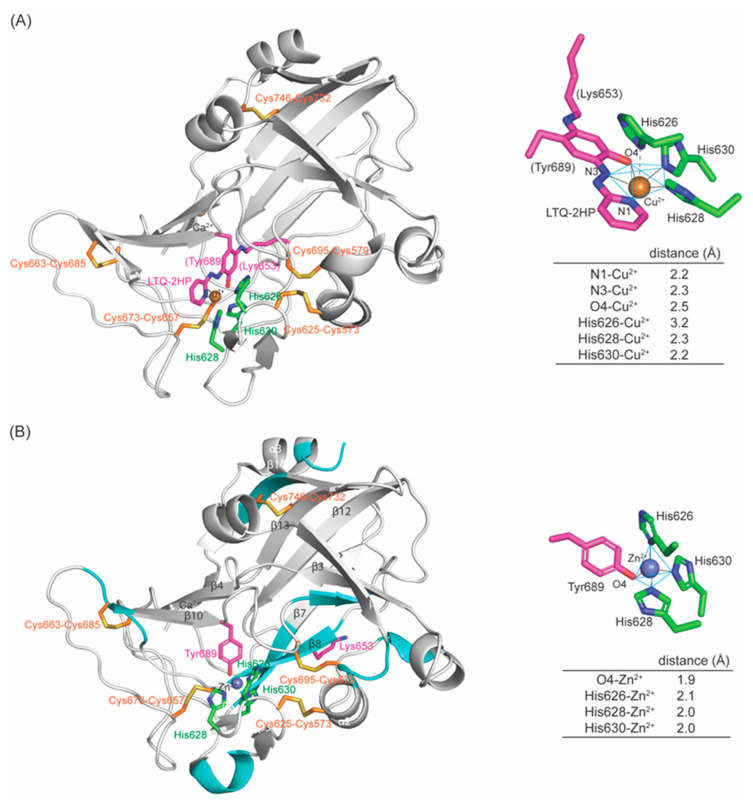
Comparison of amine oxidase domains and metal–binding sites of the 3D–modeled 2HP–inhibited LOXL2. (**A**) and the crystal structure of Zn^2+^–bound precursor (PDB:5ZE3) (**B**). Overall structures are very similar (RMSD = 1.347). In 3D–modeled structure (**A**), the cyan–colored secondary structure features in the precursor (**B**) are missing (having become loops). In the 2HP–ihibited LOXL2 (**A**), the Cu^2+^–coordination environment is a distorted square pyramidal with O4–Cu^2+^ in a Jahn–Teller axis and N1 and N3 of LTQ–2HP and His628 and His630 comprising the square bottom. In the precursor structure (**B**), Zn^2+^ is in tetrahedral coordination geometry having O4, His626, His628 and His630 at each tip of the tetrahedron. The cysteine–paring pattern of the five disulfide bonds are totally conserved in both structures.

The active site Cu^2+^ is penta–coordinated where LTQ–2HP serves as a tridentate ligand, with His628 and His630 being the remaining two ligands (Figure 10A, right panel). His626 is 3.2Å away from Cu^2+^, thus it is no longer ligating to Cu^2+^. The O4 (oxoanion) of LTQ–2HP is 2.5Å from Cu^2+^ which is 0.2–0.3Å longer than other ligands to Cu^2+^ at the Jahn–Teller axis. The X–ray crystal structure of Cu^2+^–ligated LTQ–2HP model compound revealed that Cu^2+^ is in a distorted square pyramidal geometry with an Addison parameter (τ) of 0.004, where two bridging chloride ligands likely correspond to His628 and His630 in 2HP–inhibited LOXL2 (Figure S1B) [25,30]. At each frame during MD–simulation, the τ values in the average of 0.195 ± 0.003 and the maximum of 0.387 were calculated (Figure 8Β), supporting the hypothesis that the Cu^2+^ coordination geometry is distorted square pyramidal. These results are also consistent with the distorted square pyramidal Cu^2+^ coordination geometry observed in 2HP–inhibited Y369F–ECAO (τ = 0.397) where TPQ–2HP serves also as the tridentate ligands (O4 of TPQ–2HP at the Jahn–Teller axis) and two out of the three Cu^2+^–binding site His residues serve as the remaining ligands (Figure S1A) [26]. In our modeled–structure, His626 is similarly 3.2Å away from Cu^2+^ and is not ligated to Cu^2+^.

### 2.5. Comparison of the Active Sites of 3D–Modeled 2HP–Inhibited LOXL2 and the Zn^2+^–Bound Precursor LOXL2

The overall 3D–modeled structure of 2HP–inhibited LOXL2 is superimposable to the crystal structure of the precursor with RMSD = 1.527Å for amine oxidase domains. Some secondary structures, mostly β–sheets in the precursor structure (in cyan), become shorter or absent in the 2HP–inhibited LOXL2. Besides the Cu^2+^–bound LTQ–2HP moiety, notable differences observed are (1) the position of Tyr689 (Figure 10A); (2) the conformation of the peptide Ala649–Glu659 containing Lys653 (Figure 10A,B), where C4 and C5 of His652 are in Van der Waals contact with the peptide backbone of Lys653; (3) the conformation of Phe653 with no π–π stacking interaction of Tyr689 and Phe653 (Figure 10A and Figure S2A); (4) no Van der Waals contact between Lys653 and His637 (although the conformation of His637 is totally conserved) (Figure S2B,C). In addition, there are some small differences such as (1) Cu^2+^ has shifted ~1.0Å from Zn^2+^ along with LTQ–2HP; (2) the orientation of imidazole rings of His628 and His630 that comprise the distorted square pyramidal coordination geometry where LTQ–2HP moiety serves as a tridentate ligand (Figure S2A). The 1.0Å shift of Cu^2+^ is most likely originated from LTQ–2HP ligating to Cu^2+^, and we anticipate that the unmodified LTQ in the mature LOXL2 has some motional flexibility (e.g., pivoting) in between the two conformations.

### 2.6. Molecular Modeling and MD–Simulation of the Resting Form of the Mature LOXL2

Based on the 3D–modeled 2HP–inhibited LOXL2 structure (Figure 10A), the resting form of the mature LOXL2 (e.g., LTQ–unmodified) was generated by molecular modeling and MD–simulation. Two water molecules at the equatorial and the axial positions of Cu^2+^ (Weq and Wax) were included to comprise the square pyramidal Cu^2+^–coordination geometry with His626, His628 and His630, analogous to Cu^2+^–coordination site of CAOs [37,38]. In the absence of these two water molecules, LTQ directly ligates to Cu^2+^ through its carbonyl oxygens at C4 (1.7Å) and at C5 (2.8Å) (Figure S3). A similar phenomenon was observed in a 3D–modeled resting form of human LOX, where both carbonyl oxygen of LTQ were seen directly ligated to the active site Cu^2+^ (2.2Å), but three His residues were not ligated to Cu^2+^ (≥3.7Å) [23]. The Cu^2+^–coordination geometry of the native LOX isolated from bovine aorta was reported to be very similar to that of CAOs, including at least three nitrogen ligands and additional oxygen (water ligands) [39]. Therefore, we pursued MD–simulation including two water molecules.

**Figure 11:**
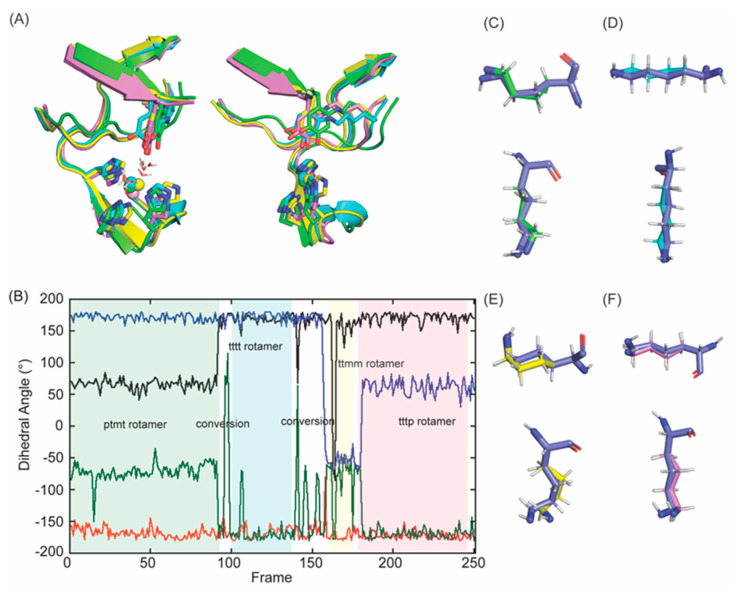
Molecular modeling and energy minimization of a 3D–model of the resting form of Δ1–2SRCR–LOXL2 in comparison to the structure obtained from MD–simulation. (**A**) An overlaid view of LTQ and Cu^2+^–binding site in four distinct configurations (frame 72: in green; frame 150: in cyan; frame 200: in yellow; frame 251: in pink in (**B**)). (**B**) Dihedral angles of Lys653 throughout the MD–simulation Χ1 (dihedral angle between N–Cα–Cβ–Cγ) (black) X2 (dihedral angle between Cα–Cβ–Cγ–Cδ) (red) X3 (dihedral angle between Cβ–Cγ–Cδ–Cϵ) (green) X4 (dihedral angle between Cγ–Cδ–Cϵ–Nζ) (blue). We observed four stable rotamers (ptmt, tttt, ttmm, tttp) among the ideal rotamers reported for Lys653: t stands for the side chain dihedral angles of 180°, p stands for the side chain dihedral angles of 65°, and m stands for the side chain dihedral angles of −65° [31]. (**C**) An overlaid view of Lys653 in frame 72 after MD–simulation (in green) with the ptmt rotamer (in slate). (**D**) An overlaid view of Lys653 in frame 125 after MD–simulation (in cyan) with the tttt rotamer (in slate). (**E**) An overlaid view of Lys653 in frame 170 after MD–simulation (in yellow) with the ttmm rotamer (in slate). (**F**) An overlaid view of Lys653 in frame 225 after MD–simulation (in pink) with the tttp rotamer (in slate).

After including two water molecules as ligands to Cu^2+^, we made the following adjustments for MD–simulation: (1) we applied a patch command to set a planar configuration between Cu^2+^ and four ligands (three His residues and Weq) by deleting angles of His626–Cu^2+^–His630 and His628–Cu^2+^–Weq and applied an improper dihedral angle to N(His626)–Cu^2+^–N(His628)–N(His630) and forced Cu^2+^–ligating atoms to remain in a plane; (2) we added a force to set a 120° angle between the Cu^2+^ and the hydrogen atoms of Weq and Wax to mimic charge repulsion. In the MD–simulation, Van der Waals interactions were excluded for short–range covalently bonded neighbors. Consequently, there was no charge repulsion for hydrogen atoms of Weq and Wax and the +2 charge of copper. The angle constraint of 120° kept the hydrogens in the correct orientation. Without these adjustments, MD–simulation resulted in s structure where LTQ was not directly ligated to Cu^2+^ (11Å away from Cu^2+^) but its Cu^2+^–coordination geometry was not precedented in literature. We observed four different stable rotamers (e.g., ptmt, ttmm, tttt, tttp) of Lys653 (Figure 11A–F) that are in the penultimate rotamer library [31] as opposed to two rotamers (pttp and mtmt) detected in 2HP–inhibited LOXL2 (Figure 6C). This indicates that the side chain of Lys653 in the resting form of LOXL2 is more flexible than that of LTQ–2HP in 2HP–inhibited LOXL2. This prediction is reasonable as LTQ is not directly associated with Cu^2+^ in the resting form but LTQ–2HP serves as a tridentate ligand to Cu^2+^ in 2HP–inhibited LOXL2. This also suggests that the active site contains enough space to adopt other stable rotamers. Among the four rotamers, the frequency of the ptmt rotamer in frames (~90 frames) (Figure 11B) suggests that it is the most stable rotamer, therefore we chose the ptmt rotamer for visualization of the MD–simulated final structure.

The maximum, minimum and average RMSF values are 0.4574, 0.0617, 0.1539 ± 0.074 nm, respectively (Figure S4A). Although the RMSD value (0.158 ± 0.002 nm) of the amine oxidase domain of the resting form is similar to that of 2HP–inhibited form (Figure 7A), the RMSD value (0.345 ± 0.002 nm) of Δ1–2SRCR–LOXL2 is ≥0.25 nm. This suggests that the amine oxidase domain is relatively stable but the two SRCR domains in the resting form have gained some flexibility when compared to the 2HP–inhibited LOXL2. The analysis of *B*–factors indicated more fluctuations observed in SRCR4 during our MD–simulation of the resting form when compared to the 2HP–inhibited form (Figure S4C). The RMSF values and Cα *B*–factors detected larger fluctuations in the SRCR4 domain (Figure S4B,C) but the amine oxidase domain and the SRCR3 domain remain very close to the original state (RMSD = 0.218 ± 0.023 nm). It should be noted that no domain–domain interaction was observed for SRCR4 and the catalytic domain, whereas SRCR3 interacts with the catalytic domain in the Zn^2+^–bound precursor structure [22,24]. Further study is necessary to understand the interactions of SRCR domains with the amine oxidase domain, but the flexibility of LTQ (Tyr689) seems to have an influence on domain–domain interactions.

### 2.7. Principle Component Analysis (PCA) and Addison Parameter (τ) Calculation for the Resting Form of Δ1–2SRCR–LOXL2

**Figure 12:**
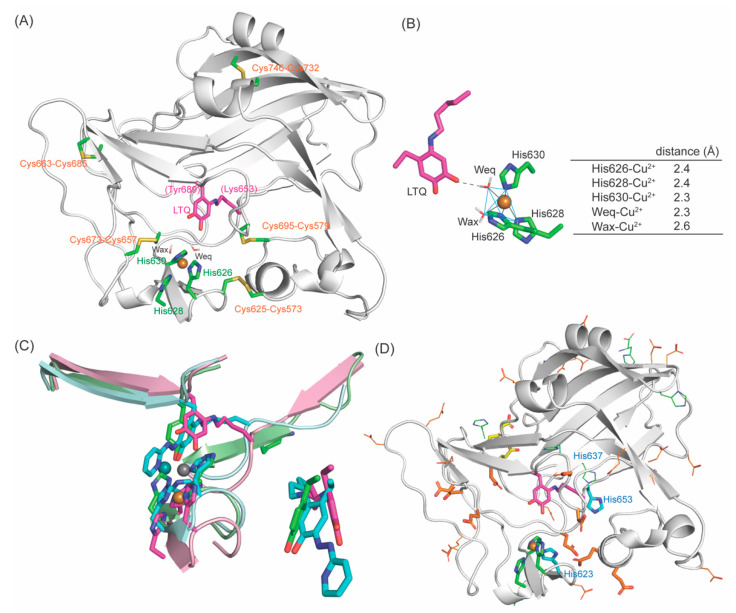
A 3D–modeled structure of the resting form of LOXL2: (**A**) The active site structure. (**B**) The Cu^2+^–coordination environment is a slightly distorted square pyramidal with Wax–Cu^2+^ in a Jahn–Teller axis and Weq, His626, His628 and His630 comprise the square bottom. O4 carbonyl of LTQ is 2.7Å from Weq. (**C**) Left: Overlay of the resting form of LOXL2 (LTQ and three His in magenta, peptide in pink, Cu^2+^ in golden sphere), 2HP–inhibited LOXL2 (LTQ–2HP and three His in cyan, peptide in pale cyan, Cu^2+^ in slate sphere) and the Zn^2+^–bound precursor (Tyr687 and three His in green, peptide in pale green, Zn^2+^ in grey sphere). Right: Overlay of Tyr687, LTQ–2HP and LTQ. (**D**) Acidic residues (Asp/Glu) and His residues in the active site. In orange stick: conserved acidic residues. In orange line: acidic residues in LOXL2 that are not conserved in the human LOX–family of proteins. In yellow stick: conserved acidic residue as a part of Ca^2+^–binding site. His623 and His653 (in cyan stick) are conserved. His637 (in blue line) is conserved in human LOX–family of proteins (Figure 4A) but not in mammalian LOXL2 (Figure 4B).

For the resting form of Δ1–2SRCR–LOXL2, the first three eigenvalues account for 50.5%, 16.4%, 5.6% of the total atomic fluctuations in the 10 ns simulation (Figure S5A). As the first two eigenvalues cover the majority of the variation, the 2D plot shown in Figure S5B was generated to visualize the majority of the variation in MD–simulation. MD–simulation produced trajectories of Cα atomic positions as a function of time and provided three representative stable states (Figure S5A,C). The feature shown in red corresponds to 0–0.64 ns of the simulation, which accounts for the mobility of SRCR4. The lack of revisiting states over the course of the simulation indicates that not all possible states have been sampled. However, the differences between the states are relatively small and it is most likely that the additional states would not have a large overall impact on this conclusion. Fluctuations were also observed at the surface of the amine oxidase domain; however, LTQ and Cu^2+^–binding site are mostly stable (Figure S4D). Importantly, the active site Cu^2+^ remains penta–coordinated with Weq, Wax, His626, His628 and His630 (Figure 12A). At each frame during MD–simulation, the τ values were calculated to be on average 0.058 ± 0.003, and the maximum to be 0.192 (Figure S4B); and these values are smaller than those calculated for 2HP–inhibited LOXL2. The average τ value is closer to zero suggesting that Cu^2+^–coordination geometry is closer to square pyramidal [40].

### 2.8. The Active Site Structure of the Resting Form of LOXL2

The MD–simulated structure of the active site of the resting form of LOXL2 is shown in Figure 12A. The active site Cu^2+^ is in slightly distorted square pyramidal geometry composed of His626, His628, His630, Weq and Wax. Wax is in the Jahn–Teller axis. LTQ has a weak interaction with Weq through the oxygen atom of the C4 carbonyl group (Figure 12B). RMSD values for overlay with MD–simulated 2HP–inhibited LOXL2 and Zn^2+^–bound precursor structure are 1.459 and 1.070, respectively, suggesting that overall structures are very similar. The noticeable differences are seen at the position of the LTQ ring and Cu^2+^–binding site (Figure 12C, left panel). In 2HP–inhibited LOXL2 and Zn^2+^–bound precursor structure, LTQ–2HP and Tyr689 serve as ligands to Cu^2+^ and Zn^2+^; and because of the ligation, Cu^2+^ and Zn^2+^ as well as His626–X–His628–X–His630 were pulled towards LTQ–2HP and Tyr689. The quinone ring of LTQ has moved ~4.2Å away from the phenyl ring of Tyr689, supporting our hypothesis that LTQ can pivot (~45°) in the active site (Figure 12C, right panel) and there is some space in the active site to accommodate this motion of LTQ. LOXL2 oxidizes peptidyl lysine residues in collagen IV, tropoelastin and PDGFRβ. These known substrates are macromolecules and the mobility of LTQ may play important roles in substrate recognition and oxidation.

## 3. Discussion

An early mechanistic study of the native LOX isolated from bovine aorta has identified a residue with p*K*_a_ = 7.0 ± 0.1 [41]. Based on the p*K*_a_ values, a His residue was proposed to be the active site base. Besides His626–X–His628–X–His630 (Cu^2+^–biding site), there are three more His residues (His623, His637 and His652) that are conserved in the LOX–family of proteins (Figure 4A), but His637 is not totally conserved in mammalian LOXL2s (Figure 4B). Judging from the locations/distance of His623 and His652 from LTQ, it is less likely that these residues function as the active site base. In CAOs, a conserved Asp residue is identified as the active site base that plays a number of critical roles in the catalytic cycle of the oxidative deamination of an amine substrate [37,42,43,44]. The active site of LOXL2 has a total of 33 acidic residues (Asp/Glu) and 16 of them are conserved in the LOX–family of proteins (Figure 12D, in orange stick), where Asp549, Glu722 and Asp724 serve as Ca^2+^–binding site (Figure 12D, in yellow stick). The remaining 13 conserved acidic residues are expected to play roles as the active site base and substrate recognition/binding.

**Figure 13:**
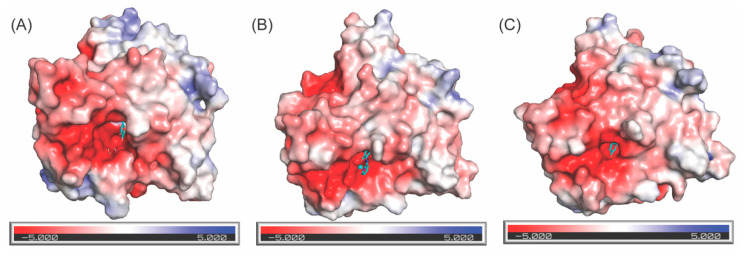
Electrostatic potential on the surface of the 3D–modeled catalytic domain of the resting form of LOXL2 (**A**), 2HP–inhibited LOXL2 (**B**) and the crystal structure of the Zn^2+^–bound precursor LOXL2 (**C**). LTQ, LTQ–2HP and Tyr689 (all in cyan) are exposed to the solvent. The acidic groove surrounding the LTQ cofactor and Cu^2+^ most likely accommodates substrate (Lys–containing peptides) binding.

The surface electrostatic potential maps of amine oxidase domains of the 3D–modeled structure of the resting form of LOXL2 (Figure 13A), 2HP–inhibited LOXL2 (Figure 13B) and the crystal structure of Zn^2+^–bound precursor (Figure 13C) were generated by PyMOL with APBS Electrostatics plug–in [45]. We found an acidic patch on the solvent accessible surface adjacent to LTQ, LTQ–2HP, the Cu^2+^–binding motif, and Tyr687; and another acidic patch on the backside of the Cu^2+^–binding motif (not shown). In all three structures, LTQ, LTQ–2HP, Tyr687, Cu^2+^ and Zn^2+^ are exposed to the surface. These acidic residues in those grooves are conserved in the LOX–family of proteins. It is most likely that a peptidyl–lysine substrate interacts with these surface–exposed acidic grooves near the active site. However, further study is necessary to investigate the mode of substrate (Lys–containing proteins) binding.

## 4. Materials and Methods

### 4.1. Molecular Modeling of the Amine Oxidase Domain of 2HP–Inhibited LOXL2

The molecular modeling of the amine oxidase domain of 2HP–inhibited LOXL2 was performed by CHARMM version 40b1 using the CHARMM36 (C36) all atom topology and parameters [32,46], based on the amine oxidase domain of the Zn^2+^–bound precursor LOXL2 (PDB: 5ZE3). The dihedral angles in the hexapeptide (His652–Lys653–Ala654–Ser655–Phe656–Cys657) containing the precursor Lys653 were replaced by those of the corresponding hexapeptide in the AlphaFold 2–predicted amine oxidase domain of LOX (https://alphafold.ebi.ac.uk/entry/P28300). The dihedral angles for the hexapeptide were edited manually into the precursor LOXL2 structure and corresponding atomic coordinated structure was rebuilt with CHARMM.

To replace Tyr689 with LTQ–2HP, the structure of TPQ–2HP was adopted from the crystal structure of Y369F–ECAO [26] with initial predictions of topology and parameters generated by CGenFF interface version 1.0.0 [47,48,49,50]. The ϵ–amino group of Lys653 was manually crosslinked to C2 of Tyr689 to generate LTQ–2HP by a use of the patching function of CHARMM. The Zn^2+^ from the precursor LOXL2 structure was then replaced with Cu^2+^ by applying the appropriate Lennard–Jones parameters [51]. The resulting preliminary structure was then subjected to energy minimization for 1600 steps, where a decreasing harmonic restraint and a position fix constraint were applied to His626, His628, His630 and LTQ–2HP, respectively. A second round of energy minimization was performed for 1600 steps with a decreasing harmonic restraint being applied to LTQ–2HP. The energy–minimized structure was then solvated in a 99Å water box where a total of 27,786 TIP3P water molecules implemented in the CHARMM force field were added [52]. Charges were balanced in the structure by adding 0.15 M NaCl to mimic physiological conditions before continuing to MD–simulation.

### 4.2. MD–Simulation of the Amine Oxidase Domain of 2HP–Inhibited LOXL2

CHARMM–GUI [33,46] was used to convert CHARMM files (structure, topology and parameter) to GROMACS format for energy minimization [53]. The model was first energy–minimized by using the steepest descent algorithm with convergence until the maximum force reaches below 1000 kJ mol^−1^ nm^−1^, and then equilibrated for 125 ps under constant pressure at 303.15 K using the Nosé–Hoover thermostat [54,55]. Production runs were conducted using GROMACS and molecular dynamics simulations were performed at 303.15 K using the Nosé–Hoover thermostat and the pressure at 1 bar using the Parrinello–Rahman coupling algorithm [56,57]. All bonds between heavy atoms and hydrogens, as well as artificial H–H bonds in the rigid TIP3P water model were constrained using the LINCS algorithm [58] with an integration time step of 2 fs. The Van der Waals interaction, Lennard–Jones potential [59], and the Smooth Particle–mesh Ewald cut–offs [60] were set to 1.2 nm. The center–of–mass motion was removed every 0.2 ps. Production steps were carried out for a total of 10 ns, trajectory frames were saved every 40 ps and all data were exported to Kaleidagraph (Version 5.0, Synergy Software, Reading, PA, USA, www.synergy.com) and analyzed.

### 4.3. Molecular Modeling of the Amine Oxidase Domain of Active LOXL2

The molecular modeling of the amine oxidase domain of the resting form of LOXL2 was performed by a similar method applied to the molecular modeling of the 2HP–inhibited LOXL2 described earlier. To generate LTQ from the LTQ–2HP motif, all atoms derived from 2HP were manually deleted and the carbonyl oxygen was added at C5. The active site Cu^2+^–coordination site was edited from that of the 2HP–inhibited LOXL2 by removal of the direct interaction between C4 carbonyl oxygen of LTQ and Cu^2+^. After energy minimization for 1600 steps, where a decreasing harmonic restraint was placed on His626, His628, and His630, we noticed that the C4 carbonyl oxygen was still directly interacting with Cu^2+^ (1.7Å). To prevent the direct interaction of C4 carbonyl oxygen and Cu^2+^, two TIP3P water molecules (Weq and Wax) were manually added as detected in the Cu^2+^–binding site of CAOs [37,38]. To compensate for the lack of nonbonded interactions between the Cu^2+^ and the two bound waters, a restraint keeping the Cu^2+^–O–H angles close to 120° were added. The resulting modeled structure was then subjected to energy minimization for 1600 steps, where a decreasing harmonic positional restraint was placed on His626, His628, His630, Weq, Wax as well as LTQ. A second round of energy minimization was performed for 1600 steps with no positional restraints applied. The energy–minimized structure was then solvated in a 99Å water box where a total of 27,947 TIP3P water molecules implemented in the CHARMM force field were added [52]. Charges were balanced in the structure by adding 0.15 M NaCl to mimic physiological conditions before continuing to molecular dynamics simulation.

### 4.4. MD–Simulation of the Amine Oxidase Domain of the Resting Form of LOXL2

The MD–simulation of the amine oxidase domain of the resting form of LOXL2 was conducted by the same methodology applied for the 2HP–inihibited LOXL2 described earlier (4.2).

### 4.5. Molecular Structure

The molecular structures presented in this manuscript were prepared by PyMOL (The PyMOL Molecular Graphics System, Version 2.0 Schrödinger, LLC, New York, NY, USA; https://www.pymol.org).

## 5. Conclusions

In this study we generated and analyzed two 3D models of LOXL2. The first was a 3D model for 2HP–inhibited Δ1–2SRCR–LOXL2, based on our solution study and the amine oxidase domains of the Zn^2+^–bound precursor Δ1–2SRCR–LOXL2 and 3D–modeled amine oxidase domain of LOX. The second was the 3D model for the resting form of Δ1–2SRCR–LOXL2. The overall structures of the amine oxidase domains of the resting form of LOXL2 and the precursor LOXL2 are very similar (RMSD = 1.070Å) and cysteine–pairing of disulfide bonds are conserved. The major difference is the secondary structure of the pentapeptide (His652–Lys653–Ala654–Ser655–Phe656), which is a loop in the former as opposed to β–sheet in the latter. This enabled the crosslink between Lys653 and DPQ689 derived from Tyr689. In comparison of LTQ, LTQ–2HP and Tyr689 in three structures, the active site has some space to accommodate motional flexibility (~45° pivoting) of the LTQ ring and the Cu^2+^–binding site is located ~5.4Å from LTQ. Both LTQ and Cu^2+^ are exposed to the surface, surrounded by acidic residues that may play important roles in substrate recognition/oxidation. This is the first 3D–modeled structure of a mature LOXL2. We believe that results of this study will advance the knowledge of the field of LOXL2 and the LOX–family of proteins that will help in the understanding of the mode of substrate/inhibitor binding and identify active site residues that are important for catalysis.

## Data Availability

The data presented in this study may be available upon request to the corresponding author (mmure@ku.edu).

## Supplementary Materials

The following supporting information can be downloaded at https://www.mdpi.com/article/10.3390/ijms232113385/s1.
