# Supplementary material for: A 3D–Predicted Structure of the Amine Oxidase Domain of Lysyl Oxidase–Like 2
Source: Int J Mol Sci. 2022 Nov 2;23(21):13385. doi: 10.3390/ijms232113385 (PMC9659206; doi:10.3390/ijms232113385)
Supplement: Supplementary file 1 [file ijms-23-13385-s001.zip › ijms-1969304-supplementary.pdf]

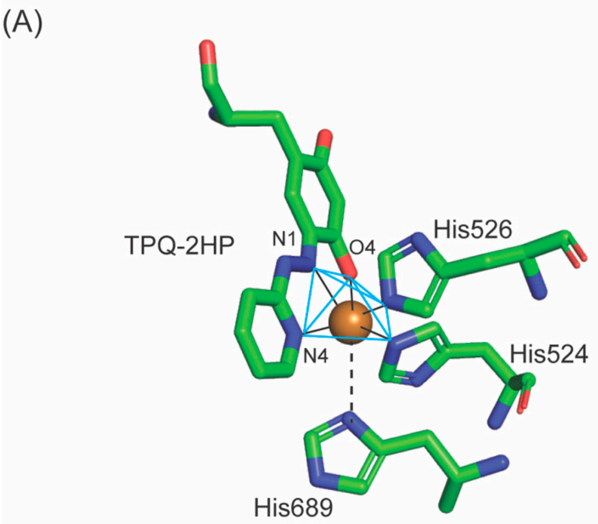

	distance (Å)
N1-Cu^{2+}	2.3
N3-Cu^{2+}	2.2
O4-Cu^{2+}	2.9
His526-Cu^{2+}	2.0
His528-Cu^{2+}	2.2
His689-Cu^{2+}	3.1

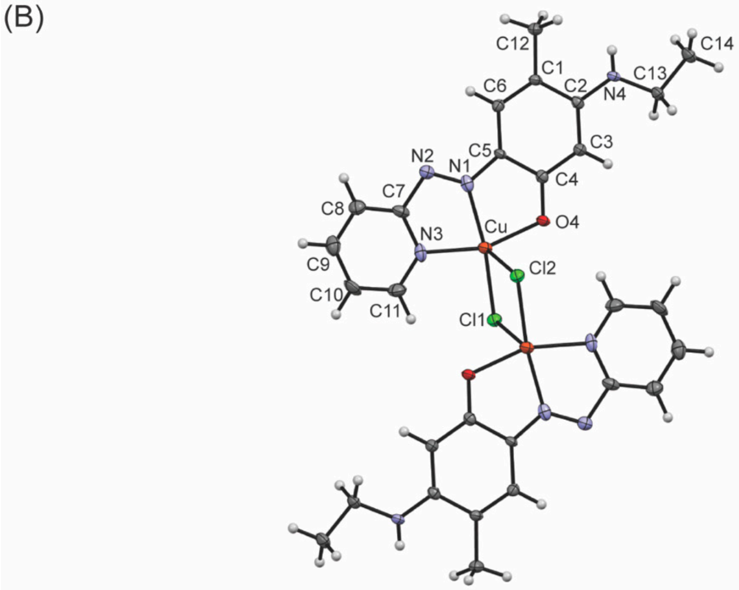

	distance (Å)
N1-Cu^{2+}	1.971
N3-Cu^{2+}	1.999
O4-Cu^{2+}	1.975
Cl1-Cu^{2+}	2.639
Cl2-Cu^{2+}	2.310

Figure S1. (A) TPQ-2HP ligated to Cu^{2+} detected in Y369F-ECAO [1]. (B) Structure of a model compound for LTQ-2HP ligated to Cu^{2+} [2, 3].

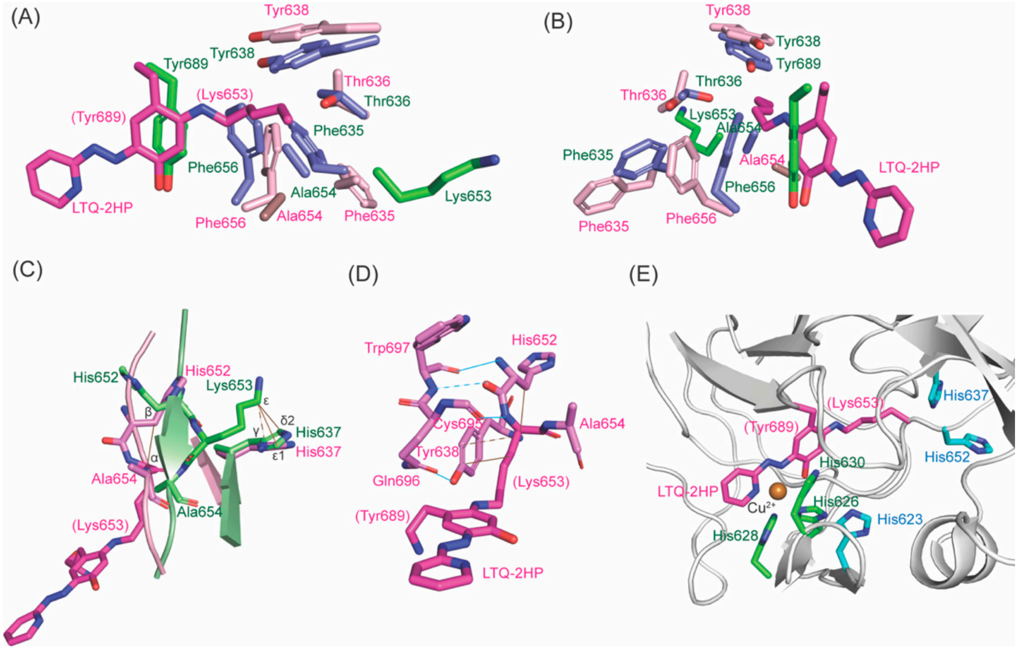

Figure S2. Van der Waals interactions observed in the crystal structure of Zn^{2+}–bound precursor (PDB:5ZE3) are absent in the 3D–modeled 2HP–inhibited LOXL2. (A) The π−π stacking interaction between Tyr689 (in green) and Phe656 (in slate) in the precursor structure is absent in the 3D-modeled structure (LTQ–2HP in magenta, Phe656 in pink). The hydrophobic patch referred to in Figure 7 (Phe635, Thr636, Tyr638, Ala654, Phe656, all in slate) is less defined in the 3D–modeled structure (the same residues in pink). (B) Another view of the area shown in (A). (C) The Van der Waals interactions (in brown lines) between Lys653 and His637 in the precursor structure (in green) are absent in the 3D–modeled structure (in pink). (D) The productive conformation of Lys653 seems to be stabilized by Van der Waals interactions (in brown lines) and hydrogen bonding interactions (in cyan lines). (E) In the 2HP–inhibited LOXL2 (in magenta), His637 is in a similar location as in the precursor, however Lys653 is in the productive conformation and thus there is no interaction with His637.

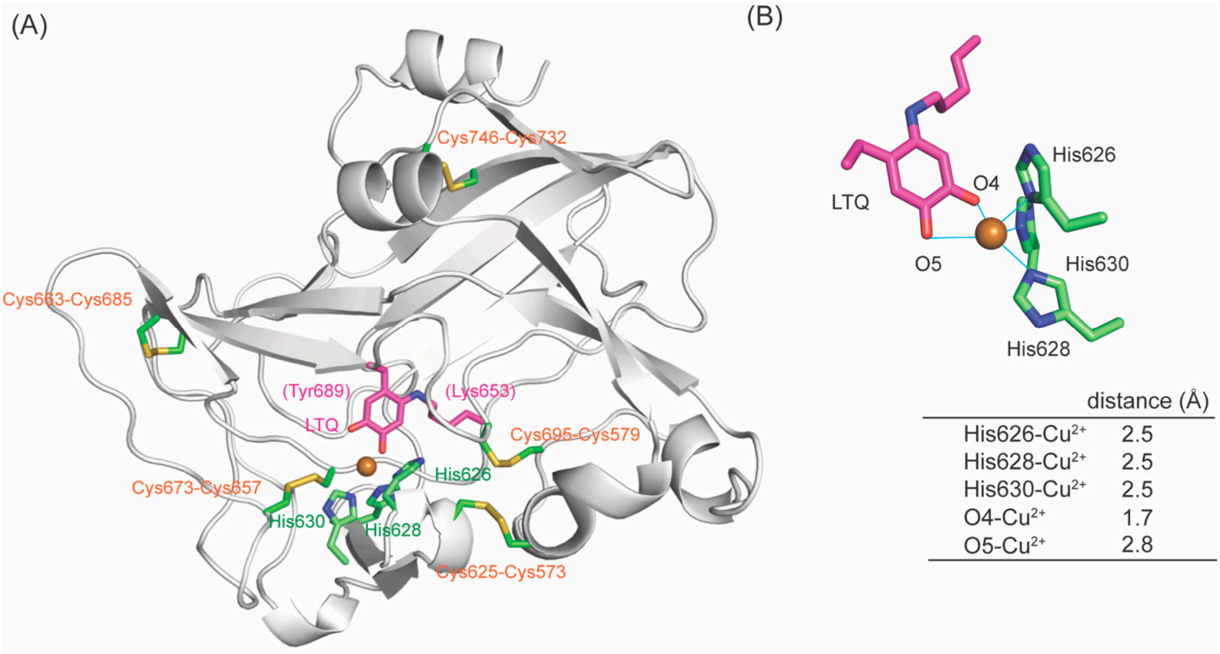

Figure S3. A 3D−modeled structure of the resting form of LOXL2 without two water ligands to the active site Cu^{2+} (see **Figure 12**) (A) The active site structure. (B) The Cu^{2+} is penta−coordinated with His626, His628 and His630 as well as two carbonyl group of the LTQ cofactor.

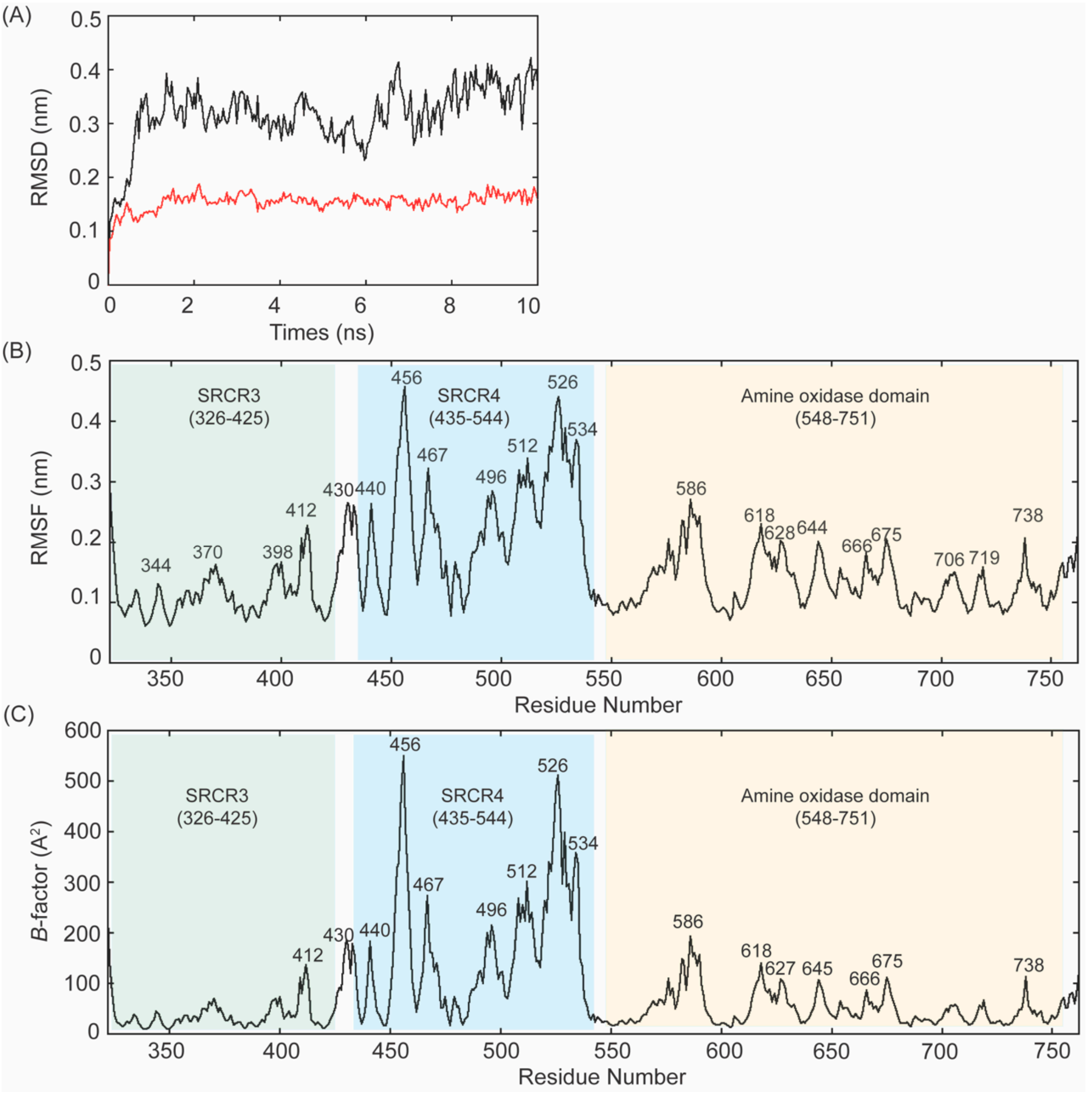

Figure S4. MD–simulation of the resting form of LOXL2. (A) Plot of RMSD for the Cα atoms of Δ1–2SRCR–LOXL2 backbone (in black) and the amine oxidase domain of LOXL2 (in red) during 10 ns of simulation. The MD–simulation for the former shows a quick increase in the RMSD value with fluctuations, stabilizing at an average of 0.31 Å, while the latter reaches to a plateau of RMSD, stabilizing at an average of 0.16 Å. (B) Plot of RMSF for the Cα atoms of Δ1–2SRCR–LOXL2 for 10 ns of simulation where fluctuations are localized at SRCR4 domain reaching to the maximum 0.457 nm. (C) Top panel: Cα *B*–factor/residue of the MD–simulated Δ1–2SRCR–LOXL2 plotted against amino acid residue number. All peaks are loops. Bottom panel: Cα *B*–factor/residue of precursor LOXL2 plotted against amino acid residue number.

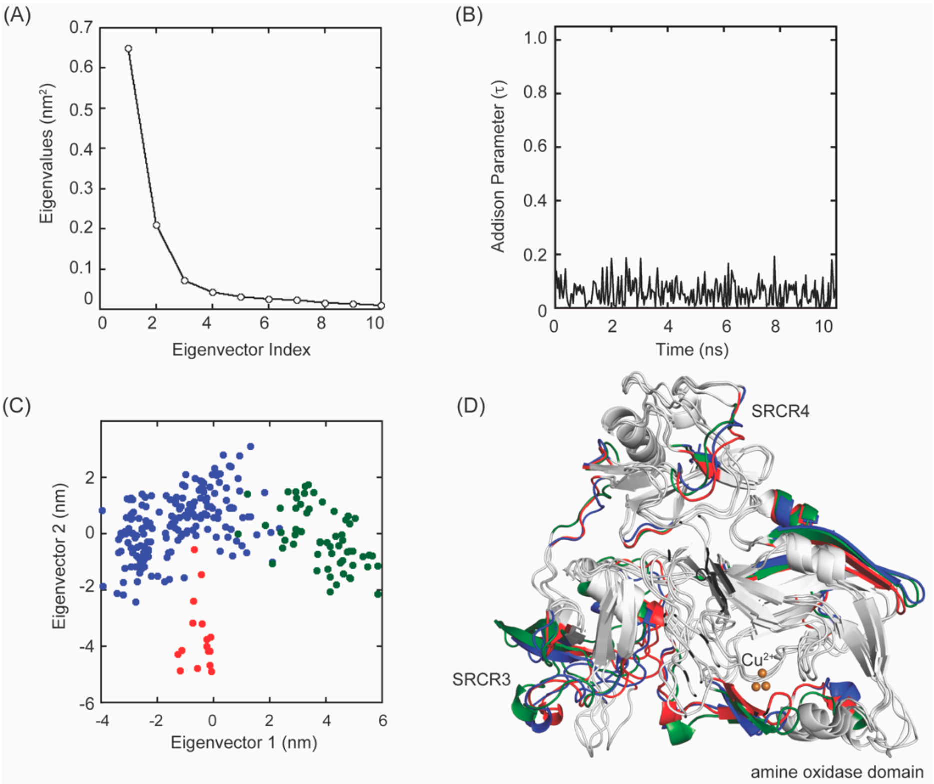

Figure S5. MD–simulation of the resting form of Δ1–2SRCR–LOXL2. (A) A plot of eigenvalues versus the first 10 eigenvector indices obtained from the covariance matrix of Cα atoms over a stable trajectory of 10 ns of MD simulation. (B) A plot of the Addison parameter (τ) values of the active site Cu^{2+} during the course of the MD simulation indicates a square pyramidal coordination geometry. (C) Projection of the motion the Cα atoms of Δ1–2SRCR–LOXL2 backbone in phase space along the first two principal eigenvectors with time 0–0.64 ns (in red), 0.68–7.64 ns (in blue), 7.68–10 ns (in green). (D) The Cα atoms corresponding to those in (C) are highlighted in the same colors.

Reference

1. Mure, M.; Kurtis, C. R.; Brown, D. E.; Rogers, M. S.; Tambyrajah, W. S.; Saysell, C.; Wilmot, C. M.; Phillips, S. E.; Knowles, P. F.; Dooley, D. M.; McPherson, M. J., Active site rearrangement of the 2-hydrazinopyridine adduct in Escherichia coli amine oxidase to an azo copper(II) chelate form: a key role for tyrosine 369 in controlling the mobility of the TPQ-2HP adduct. *Biochemistry* **2005,** 44, (5), 1583-94.
2. Meier, A. A.; Ou, S.; Day, V. W.; Mure, M., Crystal structure of (E)-5-(ethylamino)-4-methyl-2-(pyridin-2-yldiazenyl)phenol complexed with Cu (II). *submitted*.
3. Meier, A. A.; Moon, H.-J.; Sabuncu, S.; Singh, P.; Ronnebaum, T. R.; Ou, S.; Douglas, J. T.; Day, V. W.; Jackson, T. A.; Moenne-Loccoz, P.; Mure, M., Insight into the spatial arrangement of the lysine tyrosylqui-none and Cu2+ in the active site of lysyl oxidase-like 2. *submitted*.
